# The impact of systematic review of status 7 patients on the kidney transplant waitlist

**DOI:** 10.1186/s12882-019-1362-6

**Published:** 2019-05-16

**Authors:** Ashish Kataria, Madan Gowda, Brian Paul Lamphron, Kabir Jalal, Rocco C. Venuto, Aijaz A. Gundroo

**Affiliations:** 1 Division of Nephrology, University at Buffalo, Erie County Medical Center, 462 Grider Street, Buffalo, NY 14215 USA; 2 Quality and Patient Safety Department, Erie County Medical Center, Buffalo, NY USA; 3 Population Health Observatory, Department of Biostatistics, University at Buffalo, State University of New York, Buffalo, USA

**Keywords:** Kidney transplant, Waitlist, Barriers, Status 7

## Abstract

**Background:**

Increased morbidity and mortality are well documented in Status 7(inactive list) patients. Delays in transplantation secondary to prolonged periods on inactive status also negatively impacts transplant outcomes. We developed an effective system to reduce the proportion of status 7 patients on our kidney transplant waitlist. This can easily be reproduced by other transplant centers since concerns about Status 7 list size are commonplace.

**Methods:**

Meetings of a dedicated status 7 focus group were undertaken biweekly beginning in April 2016, each lasting for 1 hour or less. The group was led by a transplant physician and comprised of members from all disciplines of the kidney transplant department. Individual patient barriers to activation were systematically evaluated and action plans were developed to overcome those. The formal meetings were supplemented by updates to an electronic database accessible to all members of the team.

**Results:**

In the first 2 years of the program, we were able to activate and eventually transplant 18% of the formerly inactive patients. Forty percent of all inactive patients were removed from the waitlist due to one or more unsurmountable barriers. The median time patients stayed inactive on the waitlist was shortened from 1344 days at the start of this initiative to 581 days at the end.

**Conclusion:**

This strategy of systematic reevaluation of status 7 patients resulted in successful disposition of a substantial number of inactive patients. Further, waitlist time was reduced and transplantation expedited for the appropriate individuals. This approach could easily be adapted by other transplant centers with minimum utilization of resources.

## Background

Longer wait time on the kidney transplant waitlist is independently associated with adverse outcomes before and after transplantation, especially for the dialysis dependent patients [1]. Mortality is higher for inactive patients as compared to those active on the list [2, 3]. By the end of 2016, 30% of nearly 100,000 patients listed for kidney transplantation in the United States were inactive at any given time [4]. In addition, of the 30,000 new patient registrants on the kidney transplant waitlist in the same year, almost one third were initially inactive [4] (status 7). Referral for transplantation alone is not sufficient to improve access to transplant [5] and many patients spend almost over half of their wait time inactive on the waitlist [2, 5]. Unless intensive efforts are made to re-evaluate inactive patients on the waitlist, these patients are likely to never become active and thus are more prone to die or be delisted [6]. The barriers which exclude inactive patients from receiving organ offers could be medical, psychosocial or financial [7, 8]. In order to reduce the proportion of status 7 patients on our transplant waitlist, we developed a system to re-evaluate such patients. We speculated that our system will identify and overcome these barriers, shorten the inactive duration and consequently expedite their transplants.

## Methods

A dedicated status 7 focus group was assembled in April 2016 and met biweekly in parallel to the regular candidate selection committee. The group was led by a transplant physician and included members from all disciplines of the transplantation team including a social worker, financial coordinator, transplant office assistant, pre-transplant coordinator and the program’s quality control officer. These meetings lasted for one hour or less during which individual patient barriers to activation were discussed and intensely scrutinized in an efficient but meticulous manner. Medical, psychosocial or a financial action plans were developed and implemented for each patient to overcome one or more barrier. Typically, this would include reminder phone calls, scheduling pending diagnostic tests and consultations as necessary. Individual patient progress was tracked using an electronic list in the order of inactive duration, with dialysis dependent patients first (Table 1).

**Table 1:** Steps to reduce the inactive time (status 7) on the waitlist

Program Synopsis	
• Create a status 7 focus group led by committed individuals including a physician leader, social worker, financial coordinator, office assistant, pre-transplant coordinator and the quality control officer. • Use programs such as UNOS waitlist to identify patients and stratify them by dialysis vintage, dialysis status, cPRA and other risk factors • Hold frequent, short but focused meetings to identify individual patient’s barriers and develop action plans to overcome those. • Implementation of action plans included reminder patient phone calls, scheduling pending diagnostic tests and consultations etc. • Use the administrative assistant to communicate with the patients when appropriate • Systematic review of result of the action plans • Employ a group accessible electronic patient list to track action plans and update results between the formal meetings. • Promptly remove listed patients whose barriers are considered permanent and insurmountable	

We used a Microsoft Excel spreadsheet stored on a shared hard-drive which is accessible by all team members. The spreadsheet readily permitted the members to communicate between the meetings and quickly sort the patient database by their cPRA (calculated panel reactive antibodies), dialysis duration or age within minutes. The database has separate columns for each team member for their specific inputs. Individual members update the database between the meetings while the physician champion generated patient specific action plans during the meeting, thus making the whole process more efficient. If the review process revealed that the barriers for transplantation were definitely insurmountable, the patients were promptly informed and removed from the list.

### Statistical analysis

A descriptive analysis of the status 7 patient list 2 years after the start of program was performed where in patient demographic and listing outcomes were studied. Common reasons for inactivation or removal were classified as: Cardio-pulmonary (CP), Psychosocial (PS), Oncologic (Onc), Infection-(I), Poor functional status (F) or Miscellaneous (M).

Waitlist times separated by cohort (2016 vs. 2018) have been summarized providing mean and median (including 95% confidence intervals) times, standard deviations as well as minimum and maximum times. Histograms were generated for each cohort. Normality assumptions were tested using Shapiro-Wilk (SW) and Kolmogorov-Smirnov (KS) tests, and equality of variance was assessed using an F-Test. Finally, the Wilcoxon rank-sum test was used to compare the location of the distributions. The study was exempt from the ethics approval since it is a part of regular quality measure for the program.

## Results

The results for the first 2 years are summarized in Table 2. A total of 257 status 7 patients were evaluated between April 2016 and April 2018, of which 46 patients (18%) were able to eventually overcome one or more barriers preventing activation. Thirty nine of these 46 activated patients received a transplant during the initial 2-year period. Sixty-seven patients (26%) remained inactive.

**Table 2:** Demographic and wait list outcomes of all patients studied during the study period

Current waitlisted status	Status 7	Status 1	Died	Removed from the waitlist^a^
Number of patients, (%)	67 (26.1%)	46 (17.9%)^a^	42 (16.3%)	102 (39.7%)
Mean age (years)	51.7	54.0	60.3	60.9
Common barriers against activation (%)	CP (52), PS (30)	CP (37), PS (37), Onc (17)	CP (55), PS (21), Onc (17), I (14)	CP (46), PS (27), Onc (10) F (16)

Cardio-pulmonary *CP*, Psychosocial *PS*, Oncologic *Onc*, Infection *I*, Lack of functional status (F)

^a^39 among those received a transplant during the study period

One patient could have more than single barrier against activation

**Fig. 1:**
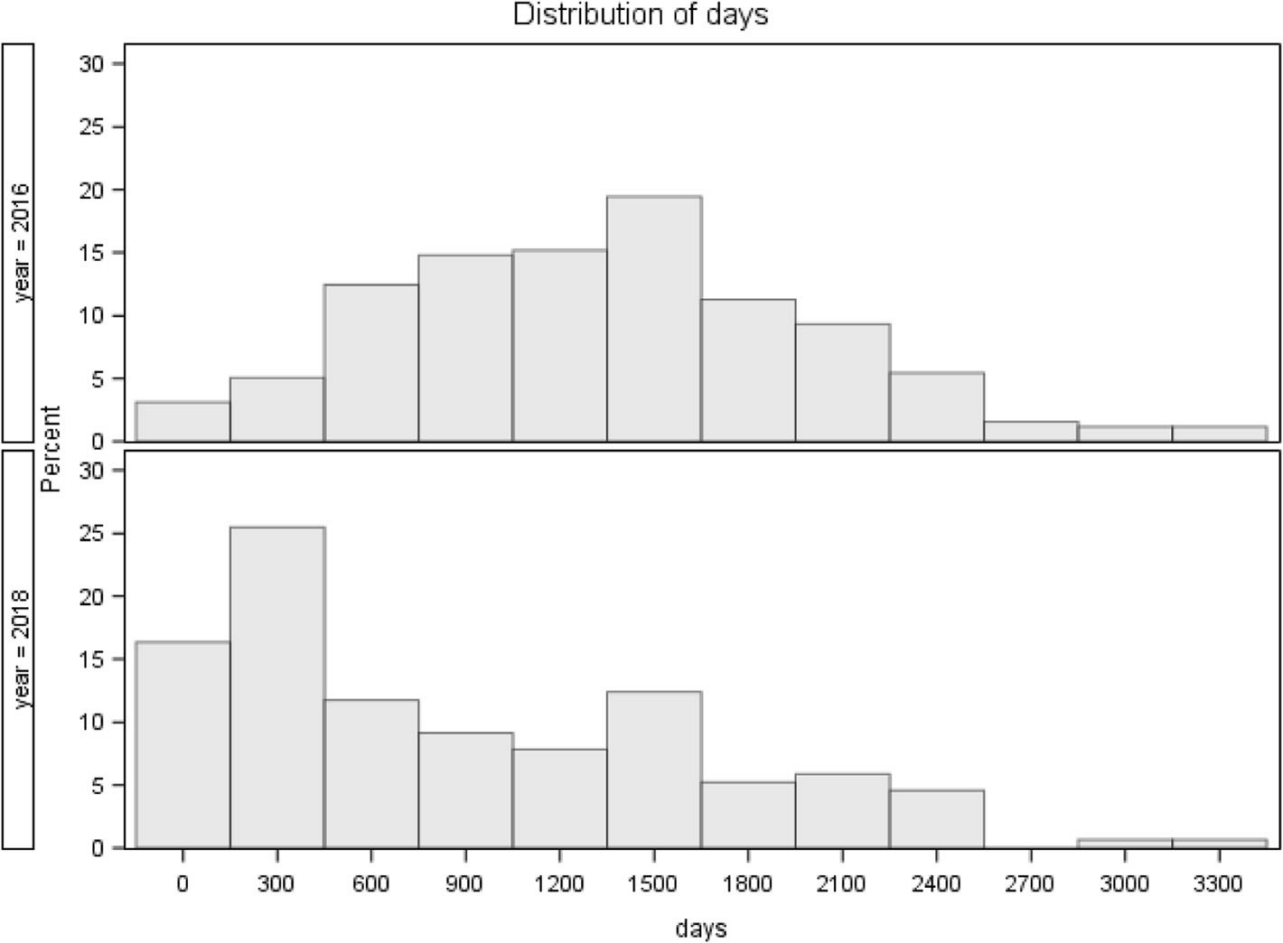
Histogram of Kidney Transplant Waitlist Times by Cohort

Forty-two (16%) inactive patients died over the 2 years while on the wait list and 102 (40%) were eventually deemed ineligible for kidney transplant and removed from the waitlist. The median inactive duration was shortened from 1344 days at the start of study to 581 days at the end. (Wilcoxon Rank Sum *P*-Value: *P* < 0.0001, Table 3). Figure 1 shows the percentage distribution of patients by wait list time over the 2-year period.

**Table 3:** Summary of Days on Kidney Transplant Waitlist

Cohort	N	Mean	Std. Dev.	95% Lower	95% Upper	Median	95% Lower	95% Upper	Min	Max
Overall	410	1160.2	738.8	1088.5	1231.9	1165	1043	1298	2	3416
2016	257	1336	669.2	1253.8	1418.2	1344	1220	1416	28	3416
2018	153	865	758.1	743.9	986.1	581	468	944	2	3411

Patients who died or those who were removed from the waitlist were older as compared to those who were activated (mean age 60.6 versus 54.0 years). These patients also spent substantially more time inactive on the waitlist as compared to those receiving a transplant (1381 versus 887 days). The mean age of status 7 patients at the start and at the end of study period was 57.2 and 52.9 years respectively.

The most common barriers against activation were cardio-pulmonary, the frequency ranging between 37 and 52% among all patient groups followed by psychosocial factors in 21–37% patients. Miscellaneous barriers included obesity, delay in patient follow up and insurance issues. In addition, 18% of patients were removed from the waitlist due to poor functional status.

## Discussion

There is a positive correlation between time spent on the transplant wait list and patient morbidity and mortality [9]. The success rate of renal allografts also declines progressively as candidate wait time increases [1, 10]. Nonetheless, a substantive portion of patients remain status 7 at the transplant centers throughout the US [4]. Individual kidney transplant programs have been urged to take steps to decrease the inactivity on waitlist not only to reduce mortality and increase access to transplantation but also to optimize utilization of transplant program resources. The American Society of Transplantation, for example, recommends annual reevaluation of high-risk patients on the waitlist [11]. Successful activation of the candidates requires vigorous efforts from the transplant center, often involving innovative and non-conventional approaches [12, 13].

In response to this need, we initiated a structured, cost efficient and easily reproduceable program to reduce the number of patients and the length of time they stayed inactive on the list (Table 1). A multidisciplinary team comprised of representatives of all elements of the transplant program was organized. Under the leadership of a transplant nephrologist, regular biweekly meetings that never exceeded an hour were held. The patients with the longest wait times were addressed first followed by those who were dialysis dependent. These groups were given the highest priority owing to their higher mortality rates. The team members meticulously reviewed each patient’s barriers and devised time sensitive steps to overcome them. Many elements of the approaches we employed could be considered components of ordinary care of potential transplant recipients. However, we are unaware that such a programmatic and systematic plan we devised is commonly employed by most transplant programs.

The barriers against activation we most frequently encountered were similar to those described by Shafi et al. [8], who noted that that cardio-pulmonary and psychosocial causes accounted for the majority of inactive patients. This status 7 focus group’s approach resulted in successful disposition of many patients along with significant reduction of wait list time. Currently, only 35% of our waitlisted patients are status 7. With this systematic multidisciplinary approach, we were able to activate and already have transplanted 15% of inactive patients over the last 2 years, with only 26% of the originally inactive patients in 2016 still remaining inactive. The mean patient age of the current status 7 list is about 4–5 years lower when compared to our 2016 list.

Of our inactive patients, 16% died on the list. These patients were older as compared to those who got transplanted or those who are still inactive. Not surprisingly, being older is associated with reduced odds of receiving a transplant even after finishing the transplant evaluation [9]. We were able to remove almost 40% of inactive patients from the wait list at the end of 2 years due to permanent barriers. These patients waited approximately 53 months on inactive status. They were also on an average 8 years older as compared to those who received a transplant or those who are still inactive. Shafi et al. [8] found that after 18 months of inactivity, reactivation was unusual. Further, timely identification of such patients provides an opportunity to develop alternate ESRD management strategies.

Since the Organ Procurement and Transplantation Network (OPTN) policy change in 2003 allowing accrual of waiting time for candidates listed as “inactive” or status 7, many chronic kidney disease patients have benefitted by accumulating wait time despite being inactive. Many of them belong to the “high risk” categories including those with advanced age, heart disease and dialysis access issues [14]. This group increases the burden of the waitlist management. Programs such as described should especially impact this high-risk category.

## Conclusion

This model of systematic reevaluation of status 7 patients results in timely disposition of a substantial number of inactive patients. Consequently, the wait list time is reduced and transplantation for the candidates identified to be appropriate is expedited. Such programs guided by a physician champion can easily be adapted by other transplant centers with relatively little utilization of transplant program’s resources, once the structure is formulated.

## Abbreviations

CP: Cardio-pulmonary
cPRA: Calculated panel reactive antibodies
ESRD: End Stage Renal Disease
F: Poor functional status
I: Infection
M: Miscellaneous others
Onc: Oncologic
OPTN: Organ Procurement and Transplantation Network
PS: Psychosocial
